# Comparison of Quality of Carbohydrate Metrics Related to Fasting Insulin, Glycosylated Hemoglobin and HOMA-IR in Brazilian Adolescents

**DOI:** 10.3390/nu14122544

**Published:** 2022-06-19

**Authors:** Camilla Medeiros Macedo da Rocha, Vanessa Proêza Maciel Gama, Amanda de Moura Souza, Edna Massae Yokoo, Eliseu Verly Junior, Katia Vergetti Bloch, Rosely Sichieri

**Affiliations:** 1 Instituto de Alimentação e Nutrição, Centro Mutidisciplinar UFRJ—Macaé, Universidade Federal do Rio de Janeiro, Av. Aluizio da Silva Gomes 50, Novo Cavaleiros, Macaé 27930-560, RJ, Brazil; 2 Instituto de Medicina Social, Universidade do Estado do Rio de Janeiro, Rua São Francisco Xavier 524, Pavilhão João Lyra Filho, 7º Andar, Rio de Janeiro 20550-900, RJ, Brazil; eliseujunior@gmail.com; 3 Instituto Federal de Educação, Ciência e Tecnologia Fluminense, Av. Souza Mota 350, Parque Fundão, Campos dos Goytacazes 28060-010, RJ, Brazil; vanessa.proeza@gmail.com; 4 Departamento de Epidemiologia e Bioestatística, Universidade Federal Fluminense, Rua Marques de Paraná 303, 3º Andar, Centro, Niterói 24030-210, RJ, Brazil; eyokoo@gmail.com; 5 Curso de Especialização em Nutrição Clínica, Instituto de Nutrição Josué de Castro, Universidade Federal do Rio de Janeiro, Av. Carlos Chagas Filho 373—Bloco J 2º Andar, Ilha do Fundão, Rio de Janeiro 21941-902, RJ, Brazil; 6 Instituto de Estudos em Saúde Coletiva, Universidade Federal do Rio de Janeiro, Avenida Horácio Macedo s/n, Ilha do Fundão, Rio de Janeiro 21941-598, RJ, Brazil; amandamoura@iesc.ufrj.br (A.d.M.S.); kbloch@iesc.ufrj.br (K.V.B.)

**Keywords:** glycemic control, intake, adolescents, glycemic index, glycemic load

## Abstract

Low glycemic index (GI) and glycemic load (GL) diets are effective for glycemic control (GC) associated with a carbohydrate-controlled meal plan. However, whether GI and GL peaks are related to GC is unknown. Objective: To compare the daily GI (DGI)/GL (DGL) and average GI (AvGI)/GL (AvGL) of meals (accounting for peaks) related to GC markers (GCM) in Brazilian adolescents. Methods: A representative national school-based (public/private) sample of students without diabetes, 12–17 years of age, was evaluated. Food intake was based on a 24 h recall. The models for complex cluster sampling were adjusted (sex, sexual maturation, age, and physical activity). Results: Of 35,737 students, 74% were from public schools, 60% girls, 17% overweight, and 8% obese. The minimum DGI and DGL were observed at lunch, with higher values at night. Fasting insulin was 1.5 times higher in overweight/obese (OW) girls, and 1.7 times higher in OW boys than in normal-weight (NW) girls. The same trend was observed for the homeostatic model assessment for insulin resistance (HOMA-IR) (OW = 2.82 vs. NW = 1.84 in girls; OW = 2.66 vs. NW = 1.54 in boys; *p* < 0.05). The daily and average metrics were greater for NW adolescents. Glycosylated hemoglobin was not associated with these metrics, except for AvGL. Insulin and HOMA-IR were associated with all metrics in NW adolescents, with greater coefficients associated with AvGL. Among overweight/obese adolescents, only GI metrics were associated (β = 0.23; AvGI and insulin) and appeared to have the best association with GCM. Conclusions: Among NW adolescents, GL is a better measure of carbohydrate quality, but for those with overweight/obesity, carbohydrate consumption is more associated with GC, probably because they eat/report small amounts of carbohydrates.

## 1. Introduction

There is consistent evidence of the protective role of a low-glycemic index (GI) diet in diabetes. For adults with diabetes, studies have clearly indicated that diets with a low GI promote better glycemic control. Both glycated hemoglobin and fasting glucose levels are reduced compared to low- and high-GI diets, and a low-GI diet also improves insulin sensitivity [1,2]. GI refers to the increase in glycemia after the intake of 50 g of available carbohydrates and characteristics of carbohydrate chain, such as monosaccharides, type of starch with amylose being less readily digested compared to amylopectin, explain differences in GI. However, the actual overall glycemic effect of foods depends on the amount of ingested carbohydrates, which are measured by the glycemic load (GL) and other components of the meal, such as fat and protein content and organic acids [3]. For patients with diabetes, both the amount and quality of carbohydrates are usually controlled.

The role of carbohydrate metrics in the prevention of diabetes, cardiovascular disease and obesity is not well understood. A meta-analysis did not show a protective effect of low GI food intake [4]. A systematic review of 21 randomized controlled trials including 2538 participants indicated that there was no convincing evidence of the effect of a low-GI diet on blood pressure, serum lipids or cardiovascular events [5].

The lack of association between the quality of carbohydrate metrics and conditions other than diabetes may be related to the greater variability in the amount of carbohydrates consumed during the day.

A better understanding of the overall impact of carbohydrate intake is to evaluate the most commonly used markers of glycemic control in relation to glycemic control. Low-GI diets are commonly used to characterize the quality of carbohydrates in the diet and their impact on glycemic blood levels, but the quantity consumed is also important, as measured by the GL [3]. More recently, high peaks of glycemic load, more precisely breakfast, have also been associated with metabolic syndrome [6]. Although the quality of carbohydrate intake has been associated with non-infectious chronic diseases, the contribution of individual meals is not clear.

Adolescents are an interesting group for testing these possibilities. In addition, insulin levels increase from childhood until the development of type 2 diabetes in adulthood [7,8,9,10], preventing insulin resistance during adolescence.

This study aimed to evaluate the association of dietary indicators of the quality of carbohydrate intake with markers of glycemic control in a large representative survey of Brazilian adolescents. We measured the GI and GL of the whole diet, as well as the average GI and GL of the individual meals. Thus, the GI of the diet, the load and peaks of GI and GL were evaluated as indicators of the quality of carbohydrate intake. Insulin, HOMA-IR and glycosylated hemoglobin were used as indicators of glycemic control.

## 2. Materials and Methods

The present study is part of the Estudo de Risco Cardiovascular em Adolescentes (ERICA), a nationwide, cross-sectional, multicenter, school-based study conducted in 2013–2014 to estimate the prevalence of risk factors for cardiovascular diseases among adolescents enrolled in public and private schools in 273 Brazilian cities with over 100,000 inhabitants [11].

### 2.1. Subjects and Response Rates

The adolescents were selected by complex sampling with 32 strata (27 Federation Units and 5 sets of municipalities with more than 100,000 inhabitants representing each of the country’s macroregions). The schools were selected in each geographic stratum with a probability of selection proportional to its size, and three classes were drawn in each school [12]. Adolescents aged 12–17 years were invited to participate in the study.

The sampling process selected 1247 schools that had a total of 114,162 students enrolled, but 10.4% of them were ineligible (215 pregnant girls, 364 with physical or cognitive disabilities and 11,256 were outside the age group studied), leaving 102,327 students. Owing to the need for fasting to analyze biochemical markers and because the blood collection was performed in schools, avoiding the displacement of adolescents to a laboratory, only students from the morning session participated in this stage. Almost three quarters (70.9%) were enrolled in the morning turn and could therefore have blood samples collected. However, only 40,732 students participated in the study. Among the reasons for the abstention of 43.8%, we can list refusals of blood collection, school absences on collection days and non-fasting.

The information contained in the ERICA questionnaire was organized into blocks, and during data processing to assess quality and consistency, each block of each adolescent was searched for certain key information that classified that block as complete or not. For the analysis of the current study, blocks named student questionnaire, 24 h recall (R24h), blood sample, anthropometry and blood pressure (data not included in this article) were used, which, combined, provided complete information on 36,956 adolescents.

Other information on the sampling design [12], the methodology adopted [11] in ERICA and the percentage of response and characterization of participants and those who refused to participate [13] can be found in previous publications.

The focus of this study was to identify directions for the prevention of type 2 diabetes; therefore, 1219 adolescents who reported having diabetes were excluded. A total of 35,737 students were analyzed, including 21,489 girls and 14,248 boys.

### 2.2. Variables and Missing Data

Fasting glucose, insulin and glycosylated hemoglobin levels were measured in fasting blood samples using the methods recommended by the Brazilian Society of Clinical Pathology, following the quality criteria for laboratory analysis [11]. The homeostatic model assessment for insulin resistance (HOMA-IR) was calculated considering the fasting glucose and fasting insulin levels. This is a recognized method for assessing glycemic control in children and adolescents [14,15,16]. Fasting insulin, glycosylated hemoglobin and HOMA-IR were used as markers of glycemic control in this study.

Some blood samples were lost during transport, storage or processing, resulting in missing data for glycated hemoglobin (female = 36; male = 16), fasting blood glucose (female = 91; male = 62), insulin (female = 84; male = 56) and HOMA-IR (female = 162; male = 114).

Food intake was assessed by one R24h using the United States Department of Agriculture Automated Multiple-Pass Method [17]. Information was collected in notebooks during face-to-face interviews conducted by trained field evaluators [11]. Ten percent of the sample answered the second R24h question.

The composition of macronutrients and fiber was calculated based on the compilation of information on the nutritional composition of food from the Family Budget Survey, 2008–2009 [18]. The amount of glycemic carbohydrates in each food was estimated by the difference between the total carbohydrates and total fibers, both measured in grams.

The GI of the majority of foods’ GI were obtained from the database of the Boden Institute of Obesity, Nutrition, Exercise and Eating Disorders and Charles Perkins Centre at the University of Sydney [19] available at http://www.glycemicindex.com/, (accessed on 1 October 2018); for the 12 regional foods, data were searched in articles [20,21,22,23,24,25]; and for 30 foods without information, the glycemic indices of similar foods in composition were used. In addition, carbonated non-sugared beverages, classified as diet or light, were assigned a GI value of zero. The same was true for high-alcohol distilled spirits, such as cachaça and brandy. Beers and drinks are not included in this group because they contain a considerable amount of carbohydrates in their composition. Meat, offal and sausages have low or no carbohydrate concentrations and therefore have zero GI.

The daily glycemic index (DGI) and load (DGL) were calculated according to the recommendations of the Food and Agriculture Organization of the United Nations/World Health Organization of 1998 [26], with the amount in grams of glycemic carbohydrate consumed from a given food multiplied by its glycemic index and weighted by the total glycemic carbohydrate consumed on the day, defining the contribution of each food. The GI of each food on the day was summed to generate the DGI. DGL considers the amount of glycemic carbohydrate consumed from a given food multiplied by its glycemic index, summed for the whole day and divided by 100.

Similarly, GI and GL were calculated for each occasion of food intake reported at R24h and the average daily glycemic index (AvGI) and average daily glycemic load (AvGL) were calculated to synthesize the GI and GL peaks during the day for each of these intake moments.

Overweight and obesity are very important conditions in the development of glycemic control [7,8,9,10]. Therefore, in the present study, we chose to conduct an analysis stratified by weight status according to BMI. We considered normal-weight (NW) adolescents with a BMI z-score < 1, overweight adolescents with a BMI z-score ≥ 1 and obese adolescents with a BMI z-score ≥ 2. [27]. For the analysis, we stratified overweight and obese adolescents in the same stratum (i.e., OW).

Sex, age and sexual maturation were associated with insulin secretion [28], and the results were adjusted for them. In addition, physical activity level has an important influence on obesity and glycemic control and was included in the adjusted model [29].

The weekly time spent on physical activity was measured using a questionnaire adapted and validated for Brazilian adolescents, containing a list of 24 activities on which adolescents responded, when they were practiced, the weekly frequency, and the time spent in each session [30]. The estimate excluded low-intensity activities, such as walking the dog or caring for children, and commuting activities, such as walking as a means of transport to school, home, or work. Finally, reports of weekly physical activity greater than 2100 min/week were considered of low quality and were therefore disregarded in the analyses, resulting in 3169 missing data records (1091 girls and 1268 boys). To classify the physical activity levels, three categories were created: inactive (0 min/week), insufficiently active (<300 min/week), and active (≥300 min/week).

### 2.3. Data Analysis

Descriptive analyses involved the calculation of means and their respective confidence intervals for continuous variables and frequencies for categorical variables. GI and GL have different dimensions, and for their effects to be comparable in linear regression, they were standardized to a distribution with a mean of zero and standard deviation. The association between GI and GL and each of the glycemic control indicators was evaluated using linear regression adjusted for the factors already described. All analyses were performed considering complex sampling using the proc survey command in SAS^®^ OnDemand for Academics available at https://welcome.oda.sas.com/ (accessed on 15 January 2022).

## 3. Results

Among girls, 7.0% were classified as obese, and 17.6% were classified as overweight. Among boys, 9.6% were classified as obese, and 17.3% were classified as overweight. The majority of the students were from public schools and lived in the capital of the state. The mean adolescent age was 14 years (Table 1).

There were no differences in the DGI and AvGI according to sex or weight status, with a mean of 59. In contrast, the DGL was higher in boys, especially in those with normal weight (195). Both boys and girls with normal weight had greater DGL and AvGL values. (Table 1). Among normal weight adolescents the average of Insulin was 8.71 mU/L for girls and 7.10 mU/L for boys. Among overweight/obese individuals, these values were 1.5 times higher in females (13.20 mU/L) and 1.7 times higher in males (12.04 mU/L).

Glycosylated Hb levels were not predicted by the glycemic control markers. Only AvGL was associated with glycosylated Hb, but the magnitude of this association was small (β = 0.006). The glycemic index of the diet measured as daily and as average values (DGL and AvGL) were the best predictors of insulin and HOMA-IR, without significant differences for average or daily among NW adolescents, but greater values of the regression coefficient for the average GI among overweight/obese adolescents. In NW adolescents, AvGL was associated with insulin levels (Table 2).

The overall composition of the diet compared with the obesity status of participants was quite similar, with the greatest difference being the lower energy intake among those who were overweight/obese (Table 3).

**Figure 1:**
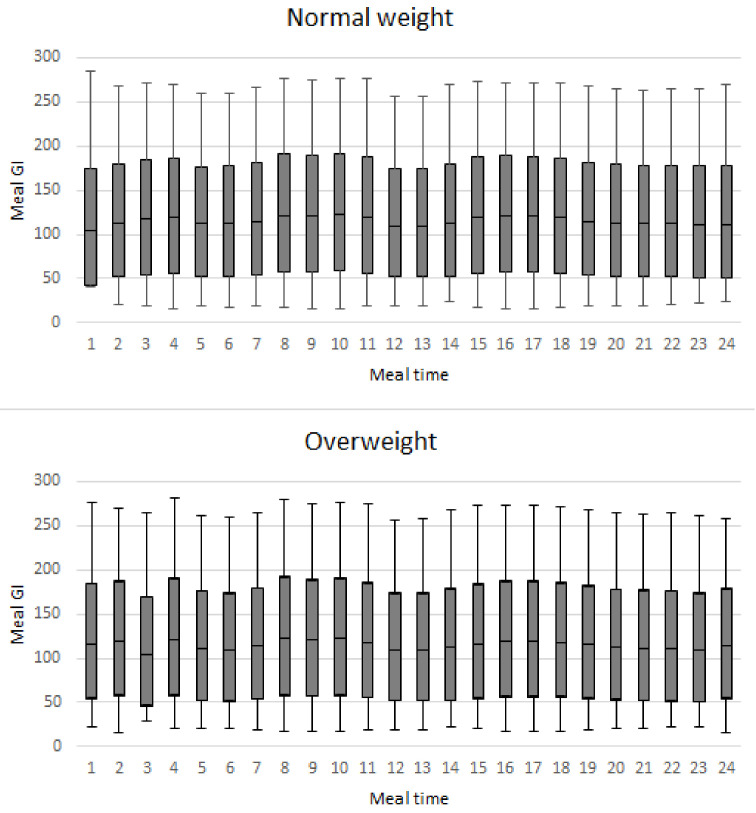
Boxplot of glycemic index of occasion meal time by weight status. Brazil 2013–2014.

Variations in GI and GL throughout the day are shown in Figure 1, with lower values of GI in occasions related to lunch, whereas intermediate intake associated with snacks had higher values without differences according to adolescents’ weight status.

## 4. Discussion

The present study provides an advance in the discussion of the effects of GI and GL on markers of glycemic control by comparing different methods of assessing these indices. The GI of chosen foods is associated with glycemic control in overweight/obese adolescents, who had a lower intake of carbohydrates, compared to NW adolescents. Differences in the weight status of adolescents and whether the index, load, average, or peak of carbohydrate intake were analyzed may explain the controversial findings in the literature.

Fasting glucose was not related to any of the carbohydrate metrics (results not shown). Although it seems controversial at first, it is necessary to consider that, in this study, we evaluated a sample of healthy adolescents. Zdravković [31] evaluated 87 obese adolescents and 17 normal-weight controls looking for symptoms of prediabetes in Belgrade. The oral glucose tolerance test showed isolated altered fasting glucose in only 13.9% of the obese group, indicating that it can be difficult to detect such an alteration.

In our study, average GI was the most important characteristic associated with insulin levels in both normal and overweight adolescents. GI meal values showed similar variation throughout the day (Figure 1) by weight status.

For mixed meals, GI peaks could be more important than the GI of individual foods. Chiavaroli [32] showed that the postprandial glycemic response of rice was greater than that of spaghetti. However, when tomato sauce and extra virgin olive oil are added to these foods, the maximum fasting glucose peak is reduced, mainly for rice. For the pesto sauce, the peaks were even smaller. The authors attributed this reduction to the addition of fat to the high-carbohydrate meal.

One study with 516 adolescents observed that GI peaks to daily mixed meals were not related, although the glycemic load of breakfast was a predictor of metabolic syndrome among girls; this finding is the only association found with only 17 cases of metabolic syndrome [6].

Cooper [33] showed that insulin increment was greater when female adolescents had a meal breakfast with a high GI compared to a low GI, with the same carbohydrate amount. Another study also showed an association between GL and metabolic syndrome in adolescence, with obesity being the most prevalent component [34]. Ojo [1] included six clinical trials that evaluated low GI diets as glycemic control markers in adults with type 2 diabetes. They concluded that a low-GI diet is more effective in controlling glycated hemoglobin and fasting blood than a high-GI diet.

According to Vega-Lopez [4], the association between GI and glycemic response with markers of glycemic control has been shown only in the strongest interventional studies, while findings from observational studies are weaker, and new diet quality metrics, such as average GI, need to be explored.

Although the GI of meals showed little variation, our findings indicate a regular pattern of intake related to the quality of carbohydrate intake. The small GI values for lunchtime are a consequence of the traditional intake of rice and beans in the Brazilian population, as shown in the last national survey [35,36,37,38].

The results of the variation in GI during the day indicate that intakes between the main meals had the greatest values, probably due to the intake of sweets, sodas and cookies as snacks. A significant intake of cookies and sugar-sweetened beverages was observed among adolescents in Brazil. The National School Health Survey (PeNSE 2012) found that 20% of 108,726 adolescents aged 14–16 years investigated regularly consume sweets and sodas [39].

A cross-sectional study of 351 children and adolescents (6–18 years of age), with and without overweight, analyzed the association between the insulinemic potential of the total diet and meals through the GI, GL, insulin index, insulin load and overweight risk. Dietary assessment was performed using a three-day food record. They found that higher insulin demand, especially at breakfast and dinner, was associated with being overweight, and night eating may be associated with eating compulsive behaviors related to obesity [40,41,42].

A limitation of the present study is that only one R24h was used, although it was based on the multiple passage method [43], which is considered a good way to stimulate memory for intake in all mealtimes [44]. In addition, measures of association using only one record are prone to sub-estimation, indicating that the effects may be greater than those observed. In addition, in large samples, it is possible to accept an isolated application when the objective is to estimate the energy and macronutrients [45].

Another possible limitation of our study is the lack of response to the blood drawn. However, Silva [13] showed no differences between participants and non-participants in relation to sex and age, but it was greater in public schools than in particular schools.

These findings suggest that carbohydrate quality metrics are associated with markers of glycemic control. The diet GI index was better at predicting insulinemia and, consequently, HOMA-IR, independent of weight status, than GL. In clinical practice, the findings show that the guidance of food consumption based on carbohydrate quality is a possibility for glycemic control, since higher GIs are highly associated with the intake of refined carbohydrates.

Encouraging healthy lifestyle habits combined with a low GI and low GL diet can also help control obesity, which is closely related to glycemic control, insulin resistance and the early development of increasing type 2 diabetes among children, adolescents and young adults. In addition, public policies that support the prevention of chronic diseases with the identification of individuals at risk of developing them, early diagnosis and individualized clinical follow-up have lower costs compared to the amount that has been spent on the treatment of type 2 diabetes and obesity worldwide.

## Figures and Tables

**Table 1:** Frequencies, means of sociodemographic, anthropometric and dietary characteristics of adolescents, by sex and weight status. Brazil, 2013–2014.

	Female *n* = (21,489)		Male (*n* = 14,248)	
Variable	Normal Weight (*n* = 16,144)	Overweight/ Obese (*n* = 5345)	Normal Weight (*n* = 10,377)	Overweight/ Obese (*n* = 3871)
Frequency (%) (95% CI)				
School Type				
Public	75.00	72.33	75.76	65.15
	(71.82; 78.19)	(68.81; 75.84)	(72.57; 78.96) ^a^	(61.09; 69,21) ^a^
Private	25.00	27.67	24.24	34.85
	(21.81; 28.18)	(24.16; 31.19)	(21.04; 27.43) ^b^	(30.79; 38.91) ^b^
Residence Area				
Capital	74.23	72.4	73.94	73.62
	(72.85; 75.60)	(70.68; 74.20)	(72.35; 75.54)	(71.62; 75.62)
Countryside	25.77	27.56	26.06	26.38
	(24.40; 27.15)	(25.80; 29.32)	(24.46; 27.65)	(24.38; 28.38)
Physical activity level ^1^				
Inactive	26.60	23.33	9.88	10.22
	(25.80; 27.41) ^c^	(22.10; 24.55) ^c^	(9.23; 10.52)	(9.21; 11.23)
Insufficiently active	33.40	32.11	26.44	28.52
	(32.58; 34.21)	(30.80; 33.42)	(25.42; 27.45)	(26.96; 30.07)
Active	40.00	44.56	63.69	61.26
	(39.17; 40.84) ^d^	(43.17; 45.95) ^d^	(62.59; 64.81)	(59.64; 62.89)
Mean (95% CI)				
Age (years)	14.74	14.47	14.72	14.38
	(14.65–14.83)	(14.37–14.58)	(14.62–14.81) ^e^	(14.27–14.48) ^e^
Daily GI	59.35	59.14	59.53	59.21
	(59.24–59.46)	(58.98–59.31)	(59.39–59.67)	(59.02–59.39)
Average GI	59.17	59.12	59.50	59.18
	(59.05–59.28)	(58.95–59.29)	(59.35–59.65)	(58.98–59.37)
Daily GL	165.22	140.61	194.86	164.94
	(163.40–167.04) ^f^	(138.30–142.91) ^f^	(192.52–197.19) ^g^	(162.05–167.83) ^g^
Average GL	37.92	34.29	45.27	40.63
	(37.55–38.29) ^h^	(33.75–34.83) ^h^	(44.77–45.76) ^i^	(39.97–41.30) ^i^
Glucose (mg/dL) ^2^	84.45	85.26	86.80	88.04
	(84.25–84.66) ^j^	(84.95–85.58) ^j^	(86.57–87.03) ^k^	(87.71–88.37) ^k^
Glycosylated hemoglobin (%) ^3^	5.33	5.38	5.40	5.42
	(5.33–5.34) ^l^	(5.37–5.39) ^l^	(5.39–5.41)	(5.40–5.43)
Insulin (mU/L) ^4^	8.71	13.20	7.10	12.04
	(8.59–8.84) ^m^	(12.91–13.48) ^m^	(6.99–7.22) ^n^	(11.74–12.34) ^n^
HOMA-IR ^5^	1.84	2.82	1.54	2.66
	(1.81–1.87) ^o^	(2.75–2.89) ^o^	(1.52–1.57) ^p^	(2.58–2.73) ^p^

Missing data: ^1^ Physical activity level: female = 1091; male = 1268; ^2^ Fasting glucose: female = 91; male = 62. ^3^ Glycosylated hemoglobin: female = 36; male = 16. ^4^ Insulin: female = 84; male = 56. ^5^ HOMA-IR: female = 162; male = 114. ^a–p^ *p*-value < 0.05 (Equal letters identify the comparison group in which statistical relevance occurs).

**Table 2:** Regression coefficients of standardized values of glycemic index (GI) on measures of glycemic control, by weight status. Brazil, 2013–2014 ^1^.

Normal Weight						
	Glycosylated Hemoglobin		Insulin		HOMA-IR	
	ß	*p*-Value	ß	*p*-Value	ß	*p*-Value
Daily GI	0.000	0.883	0.091	0.002	0.021	0.002
Average GI	0.001	0.603	0.089	0.002	0.019	0.005
Daily GL	0.004	0.074	0.057	0.059	0.012	0.082
Average GL	0.006	0.011	0.124	<0.0001	0.029	<0.0001
**Overweight/Obese**						
	**Glycosylated Hemoglobin**		**Insulin**		**HOMA-IR**	
	**ß**	***p*-Value**	**ß**	***p*-Value**	**ß**	***p*-Value**
Daily GI	−0.001	0.843	0.162	0.030	0.034	0.049
Average GI	0.003	0.3561	0.229	0.001	0.051	0.002
Daily GL	0.001	0.746	−0.084	0.308	−0.026	0.168
Average GL	−0.003	0.544	0.072	0.315	0.018	0.278

^1^ linear regression adjusted for age, sex, self-evaluated sexual maturation and physical activity (inactive/insufficiently active/active).

**Table 3:** Energy intake and nutrients by weight status. Brazil, 2013–2014.

	Normal Weight (*n* = 26,521)		Overweight/Obese (*n* = 9216)	
	Mean (95% CI)	% Total Energy	Mean (95% CI)	% Total Energy
Energy (kcal)	2372	-	2059	-
	(2351–2393)		(2034–2084) *	
Total carbohydrate (g)	316	53	271	52
	(313–319)		(267–274) *	
Glycemic carbohydrate (g)	297	50	254	49
	(295–300)		(251–258) *	
Glycemic carbohydrate from food (g)	274	46	235	45
	(272–277)		(232–238) *	
Glycemic carbohydrate from added sugar (g)	23	4	20	4
	(22–24)		(19–20) *	
Fiber (g)	19	3	16	3
	(19–19)		(16–17) *	
Protein (g)	93	16	84	16
	(92–94)		(83–85) *	
Lipids (g)	83	31	72	31
	(82–83)		(71–73) *	

* *p*-value < 0.05.

## Data Availability

The data presented in this study are available on request from the corresponding author. The data are not publicly available due to the difficulty in identifying the 4 different databases and identifying the variables’ names. All codebooks are in Portuguese.
